# Respiratory function and breathing response to water‐ and land‐based cycling at the matched oxygen uptake

**DOI:** 10.14814/phy2.15475

**Published:** 2022-09-18

**Authors:** Daisuke Hoshi, Marina Fukuie, Tatsuya Hashitomi, Takashi Tarumi, Jun Sugawara, Koichi Watanabe

**Affiliations:** ^1^ Doctoral Program in Sports Medicine, Graduate School of Comprehensive Human Sciences University of Tsukuba Tsukuba Ibaraki Japan; ^2^ Human Informatics and Interaction Research Institute National Institute of Advanced Industrial Science and Technology Tsukuba Ibaraki Japan; ^3^ Faculty of Health and Sports Sciences University of Tsukuba Tsukuba Ibaraki Japan

**Keywords:** breathing pattern, respiratory muscle training, water‐based cycling

## Abstract

The impact of underwater exercise on respiratory function remains unclear when its metabolic rate is matched with exercise performed on land. Therefore, we compared the breathing responses and respiratory function during and after water (WC)‐ and land (LC)‐based cycling performed at the matched oxygen uptake (VO_2_). Twelve healthy men performed 15 min of incremental WC and LC on separate days. During WC, participants cycled continuously at 30, 45, and 60 rpm (stages 1, 2, and 3) for 5 min each. During LC, participants cycled at 60 rpm for 15 min while wattage was increased every 5 min and adjusted to match VO_2_ to the WC condition. Breathing patterns during cycling and spirometry data before and after cycling were collected. VO_2_ during WC and LC was similar. Respiratory rate (WC: 27 ± 3 vs. LC: 23 ± 4 bpm, *p* = 0.012) and inspiratory flow (WC: 1233 ± 173 vs. LC: 1133 ± 200 ml/s, *p* = 0.035) were higher and inspiratory time (WC: 1.0 ± 0.1 vs. LC: 1.2 ± 0.2 s, *p* = 0.025) was shorter at stage 3 during WC than LC. After WC, forced vital capacity (*p* = 0.010) significantly decreased while no change was observed after LC. These results suggest that at similar metabolic rates during WC and LC, breathing is slightly shallower during WC which may have chronic effects on respiratory muscle function after multiple bouts of aquatic cycling. Underwater exercise may be beneficial for respiratory muscle rehabilitation when performed on a chronic basis.

## Graphical abstract

At similar metabolic rates during WC and LC, breathing was slightly shallower during WC which may have chronic effects on respiratory muscle function after multiple bouts of aquatic cycling.

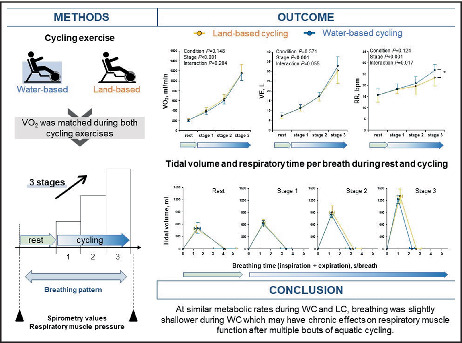

## 1 INTRODUCTION

Exercise plays an important role in respiratory rehabilitation because it can improve quality of life (QOL) and exercise tolerance and reduce dyspnea in patients with respiratory disease (Dowman et al., 2017; McCarthy et al., 2015; Wedzicha et al., 1998). However, exercise on land could increase joint pain in lower limbs in older adults, which subsequently may decrease motivation to continue exercise (Suomi & Collier, 2003). On the other hand, water‐based exercise reduces the gravitational load on lower limb joints due to buoyancy and is potentially a more attractive modality for people with limited exercise capacity (Harmer et al., 2009; Suomi & Collier, 2003). In addition, elevated hydrostatic pressure during water immersion increases venous return, which decreases lung compliance (Dahlbäck et al., 1978) and causes vertical displacement of the diaphragm (Agostoni et al., 1966). Therefore, water‐based exercise may be more effective than land‐based exercise for training respiratory muscles at the same exercise workload.

Underwater exercise may cause greater fatigue of the respiratory muscle than dryland exercise. Yamashina et al. reported that respiratory muscle strength decreased immediately after both water‐ and land‐based exercise performed at 60% peak oxygen uptake (VO_2peak_) and further that the magnitude of decrease was greater after water‐based exercise than the land‐based exercise (Yamashina et al., 2016). These observations suggest that low‐intensity underwater exercise may elicit respiratory muscle adaptations comparable to high‐intensity land‐based exercise because of increased work of breathing associated with submersion. Therefore, it is necessary to study the breathing responses at low to moderate exercise intensities in both conditions.

Comparing the physiological responses to aquatic and land‐based exercise has been challenging because the intensity of aquatic exercise varies with water resistance which depends on each individual physical characteristics and walking stride and steps. In recent years, a water stationary bicycle (WSB) has widely been used to investigate the effect of underwater exercise by controlling pedaling speed and resistance. Using WSB, several studies have reported cardiovascular responses and energy expenditure (Bréchat et al., 2013; Garzon et al., 2015; Garzon et al., 2017; Sheldahl et al., 1987). However, only a few studies have compared the ventilatory response during exercise in water and on land under the matched metabolic rate conditions (Ayme et al., 2015; Bréchat et al., 1999; Bréchat et al., 2013; Sheldahl et al., 1987). In addition, postexercise respiratory function may be altered by exercise in water due to the lung expansion to overcome the elevated hydrostatic pressure. Among those previous studies, there were inconsistent observations on the ventilatory response during underwater exercise. Some have reported no change in breathing patterns between the land‐ and water‐based exercises performed at moderate‐exercise intensity (Bréchat et al., 1999; Dionne et al., 2017). The others have reported that respiratory rate (RR) changes at low‐to‐high intensity, but tidal volume did not change at low‐to‐moderate intensity (Sheldahl et al., 1987), or that low‐intensity underwater exercise changed both RR and tidal volume (Ayme et al., 2015). Therefore, further investigations are required to clarify the effect of water‐based exercise on respiratory function. If RR and tidal volume change, other parameters such as breathing time and flow during inspiratory and/or expiratory phases also need to be examined.

During land‐ and water‐based cycling (LC and WC, respectively), matching both the external work output and metabolic rate simultaneously is difficult because water resistance exerted on the exercising legs can change with pedaling speed due to the viscoelasticity of water. Therefore, in this study, we decided to match metabolic rate while pedaling speed or external work output was different in both conditions. The purpose of this study was to compare the respiratory function and breathing responses during LC and WC performed at low‐to‐moderate intensities with the matched metabolic rate. Based on the findings from the previous studies (Ayme et al., 2015; Sheldahl et al., 1987), our primary hypothesis was that tidal volume and breathing time would be lower during WC than LC, when they were performed at the matched metabolic rate. Secondarily, we hypothesized that WC would decrease respiratory function and muscle pressure at postexercise compared with LC.

## 2 MATERIALS AND METHODS

### 2.1 Ethics statement

Participants were randomly recruited at the University of Tsukuba. All participants gave informed consent before participation. This study was performed in accordance with the latest standards set forth by the *Declaration of Helsinki*, except for registration in a database. All experimental procedures were approved by the Research Ethics Committee of the University of Tsukuba.

### 2.2 Participants

Twelve healthy men (mean age of 24 ± 1 years, the height of 174.8 ± 4.3 cm, the weight of 68.1 ± 5.7 kg, body mass index of 22.3 ± 1.6 kg/m^2^, VO_2peak_ of 39.8 ± 4.1 ml/kg/min) were studied. None of them were taking medication or had overt cardiovascular, respiratory, or neuromuscular disease or a history of smoking as assessed by a medical history questionnaire.

### 2.3 Experimental design

Participants visited the laboratory three times and had the following measurements on each day: (1) VO_2peak_ with graded exercise test, (2) WC, and (3) LC. The order of WC and LC was not randomized because the metabolic rate recorded during WC (day 2) was used to match the rate during LC (day 3). Participants were instructed to fast for a minimum of 3 h and to refrain from vigorous exercise, alcohol consumption, and caffeine intake for at least 24 h before each visit. All visits were separated by at least 72 h.

#### VO_2peak_ measurement (day 1)

VO_2peak_ testing was conducted on a semi‐recumbent bike (Corival recumbent, Lobe BV). A breath‐by‐breath expiratory gas analyzer (Aero monitor, MINATO) was used to assess VO_2_ and carbon dioxide production during exercise. The workload started at 60 W and increased by 20 W every 2 min. After reaching 160 W, the workload was increased by 10 W until two of the following criteria were met: (1) 95% heart rate reserve (95% HR reserve) calculated from the age‐predicted maximum; 95% HR reserve = (age‐predicted maximum [i.e., 220 − age] − rest HR) × 0.95 + rest HR (Karvonen et al., 1957), (2) respiratory exchange ratio greater than 1.2, (3) unable to maintain cycling at 60 ± 5 rpm, or (4) rate of perceived exertion higher than 18. VO_2peak_ was defined as the highest VO_2_ measured over >30 s during the last stage of testing.

After the maximal graded exercise test, participants took a break and practiced the spirometry and maximal respiratory muscle pressure measurements to familiarize themselves with the actual testing on days 2 and 3.

#### WC (day 2)

**FIGURE 1:**
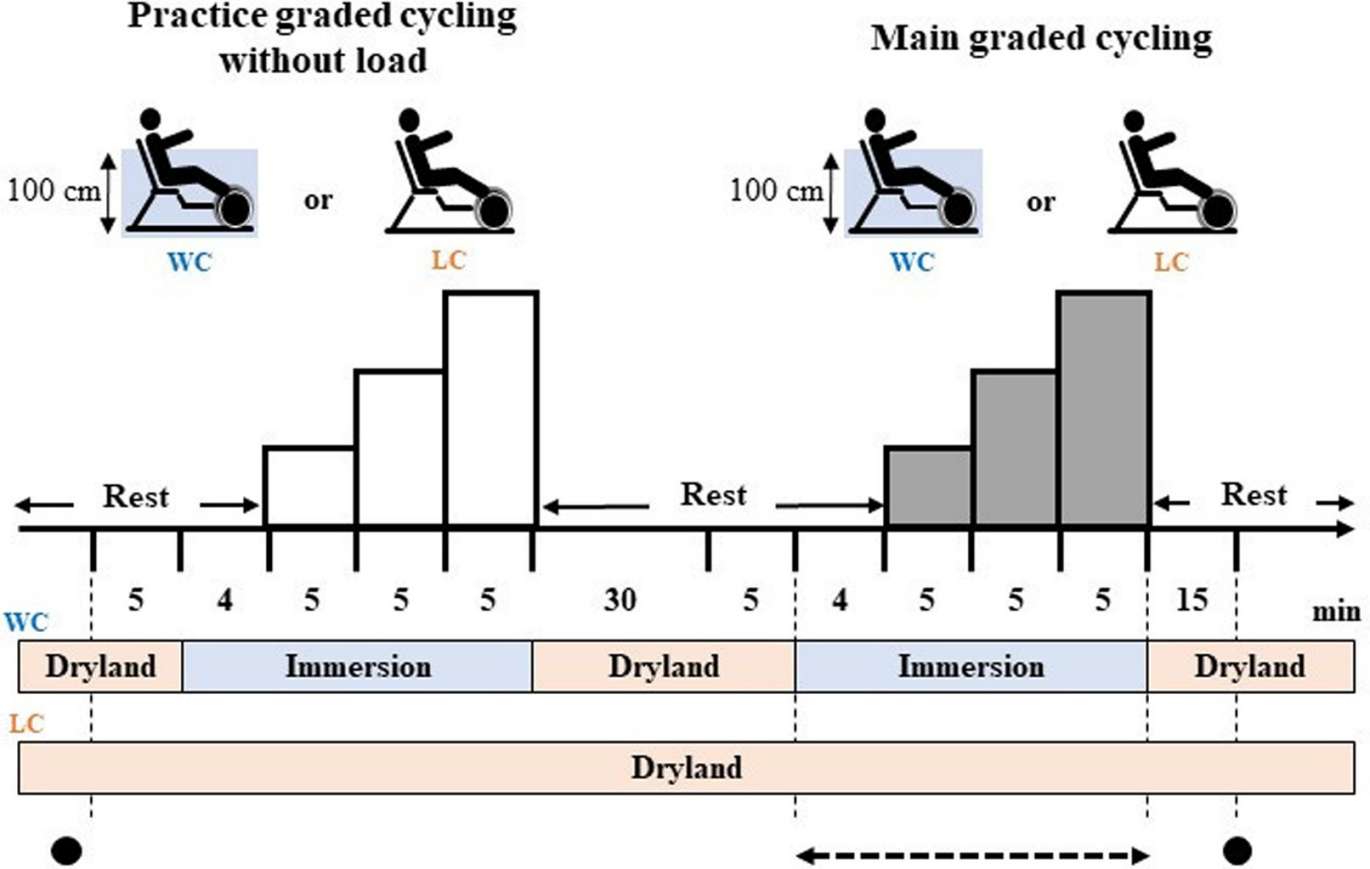
Experimental protocol of land (LC)‐ and water (WC)‐based cycling. LC and WC were conducted after practice in each condition. During WC, the water temperature was thermoneutral (31–32°C) and the depth was set to the xiphoid appendix level for each participant (approximately 100 cm). Black circles indicate the time point at which spirometry and respiratory muscle pressure were measured. The dotted arrow indicates the time point at which metabolic rate and breathing pattern were measured. WC, water‐based cycling; LC, land‐based cycling.

Figure 1 depicts the LC and WC protocols. In the beginning, participants practiced cycling for 15 min while the cadence was increased by 15 rpm every 5 min without load. After practice, participants took at least 30 min of rest as a washout period. In the WC condition, participants rested in the seated position on land for 5 min. Then, they submersed to thermoneutral water (31–32°C), sat on an underwater stationary semi‐recumbent bicycle (Hydrorecline, H_3_Oz company) for 4 min, and started cycling. WC condition consisted of three stages of continuous exercise. At each stage, participants cycled at 30, 45, and 60 rpm (stages 1, 2, and 3) for 5 min. The cadence was controlled using a metronome. Throughout WC, three plastic paddles measuring 7 cm × 22 cm × 0.5 cm were attached to the rear wheel to increase the pedaling resistance. Water depth was approximately 100 cm set to the xiphoid appendix level for each participant.

#### LC (day 3)

Participants rested in the seated position on land (room temperature: 25–26°C) for 5 min and rested for another 4 min on a semi‐recumbent bicycle (Corival recumbent, Lode BV) to match the WC condition. After resting, participants cycled at 60 rpm continuously for 15 min on the semi‐recumbent bicycle. At the beginning and every 5 min during cycling, wattage was adjusted to match VO_2_ with that recorded during each stage of the WC condition. To quickly find the right wattage which generates VO_2_ matched with WC, the relation between wattage and breath‐by‐breath changes in VO_2_ recorded on day 1 during VO_2peak_ testing was used as a reference. Using the information, it took approximately 3 min to achieve the steady‐state VO_2_ matched to the WC condition in all subjects, although WC and LC could not be performed in random order. To minimize systematic error associated with fatigue, we had more than 1 week of a wash‐out period between both conditions.

The hip joint angle between the lower and upper bodies was ~130° in both the LC and WC conditions when a subject sat on a semi‐recumbent bicycle with his feet placed on both pedals and one of them extended. A pedaling cadence was set below 60 rpm for the following reasons. First, we wanted to have each participant exercise below AT because our participants had various fitness levels, and to standardize the exercise protocol for all of them, we decided to use a slower pedaling cadence. Moreover, buoyancy made it difficult to maintain a stable posture when a pedaling cadence was increased above 70 rpm.

### 2.4 Measurements

#### 2.4.1 Metabolic rate and body temperature

VO_2_, %VO_2peak_, carbon dioxide production (VCO_2_), and respiratory exchange ratio (RER) were continuously recorded by the expiratory gas analyzer system in a breath‐by‐breath manner (Aero monitor, MINATO) during 4 min of rest and 15 min of cycling exercise in both conditions. Data reported are the average of the last 30 s during each stage. The last 30 s was chosen during pilot studies because all metabolic and breathing parameters reached a steady state with visual confirmation. Heart rate (HR), the original Borg's rating of perceived exertion 6–20 scale (RPE) (Borg, 1990), and tympanic temperature were intermittently measured at 2‐ and 4‐min during rest and each exercise stage and averaged.

#### 2.4.2 Breathing pattern

Ventilation (VE), RR, total breathing time (Ttot), tidal volume during inspiratory and expiratory phases (V_TI_ and V_TE_, respectively), inspiratory and expiratory times (Ti and Te, respectively), V_TI_/Ti and V_TE_/Te (inspiratory and expiratory flow, respectively), and end‐tidal CO_2_ (PETCO_2_) were continuously recorded by the expiratory gas analyzer system in a breath‐by‐breath manner (Aero monitor, MINATO). For data analysis, these variables were averaged over the last 30 s of the resting and cycling conditions. The timing of data collection was standardized for all participants. The expiratory gas analyzer was calibrated before each experiment according to the manufacturer's instructions.

#### 2.4.3 Respiratory function and maximal respiratory muscle pressure

Respiratory function and the maximal respiratory muscle pressure were measured before practicing cycling exercise and 15 min after cycling on dryland, and the magnitude of change between before and after cycling was calculated. Respiratory function was measured with spirometry (Chestgraph HI‐105; CHEST). Forced vital capacity (FVC), forced expiratory volume in 1 s (FEV_1_), FEV_1_/FVC, and peak expiratory flow (PEF) were measured. In accordance with the guidelines of the American Thoracic Society/European Respiratory Society (Miller et al., 2005), participants performed at least three forced respirations, from the total lung capacity to residual volume, with 2 min intervals between them, and the highest value was used for analysis. The maximal respiratory pressure was measured by a manometer (Chestgraph HI‐801; CHEST). Based on the procedures described by the American Thoracic Society/European Respiratory Society (American Thoracic Society/European Respiratory Society, 2002), the maximal expiration and inspiration were performed at the total lung capacity and the residual volume, respectively. The expiration and inspiration were maintained for 3 s and measured three times with 2 min intervals. The highest value was used for analysis as the maximal expiratory muscle pressure (PEmax) and the maximal inspiratory muscle pressure (PImax), respectively.

### 2.5 Samples size estimate

The sample size was calculated using the G*Power software. The following values were used according to a previous study (Ayme et al., 2015) that investigated breathing patterns during low‐intensity exercise in water and in dryland (i.e., effect size: 0.5; α‐level: 0.05; statistical power: 0.95; the number of groups: 2 [LC and WC]; the number of measures: 4 [rest, stages 1, 2, and 3]; considering the refusal of subjects: 2). Therefore, the sample size required for this study was estimated as 12 participants.

### 2.6 Statistical analysis

Data are presented as mean ± standard deviation (SD). All statistical analyses were performed using SPSS statistics 26.0 for Windows (IBM Inc). All breathing variables were analyzed using a two‐way analysis of variance (ANOVA) for repeated measures with condition (LC and WC) and stage (rest, stage 1, stage 2, and stage 3). Similarly, the spirometry and respiratory muscle pressure data recorded at pre‐ and postexercise were also analyzed using two‐way ANOVA for repeated measures, including time (before and after cycling) and condition (LC and WC). When a significant interaction was found, *post hoc* multiple pairwise comparisons were performed with the Bonferroni correction. *p* values of <0.05 were considered statistical significance.

## 3 RESULTS

### 3.1 Metabolic rate and body temperature

Table 1 shows metabolic rate and body temperature results during rest and 15 min of cycling in both conditions. VO_2_, %VO_2peak_, VCO_2_, RER, HR, and body temperature increased and showed the main effect of stage (all *p* < 0.05), whereas the condition and interaction effects were not significant (all *p* > 0.05). The ergometric wattage during LC increased (stage 1: 12.9 ± 3.8 W, stage 2: 44.6 ± 11.4 W, and stage 3: 105.0 ± 16.3 W).

**TABLE 1:** Metabolic rate and body temperature during rest and cycling exercise in water and on land

Stage	LC	WC	*p* value (ANOVA)		
			Condition	Stage	Interaction
Exercise load					
Rest	–	–	–	–	–
Stage 1	13 ± 4 w	30 rpm			
Stage 2	45 ± 11 w	45 rpm			
Stage 3	105 ± 16 w	60 rpm			
VO_2_, ml/min					
Rest	209 ± 39	219 ± 36	0.148	<0.001	0.204
Stage 1	403 ± 56	380 ± 57			
Stage 2	637 ± 90	606 ± 70			
Stage 3	1156 ± 182	1146 ± 143			
VO_2_, ml/kg/min					
Rest	3.1 ± 0.5	3.2 ± 0.5	0.101	<0.001	0.187
Stage 1	5.8 ± 0.7	5.6 ± 0.8			
Stage 2	9.4 ± 0.8	8.9 ± 0.7			
Stage 3	17.1 ± 2.1	16.9 ± 1.6			
%VO_2peak_, %					
Rest	7.8 ± 1.4	8.2 ± 1.4	0.115	<0.001	0.198
Stage 1	15.2 ± 2.5	14.3 ± 2.6			
Stage 2	23.9 ± 3.4	22.7 ± 3.1			
Stage 3	43.5 ± 7.6	42.9 ± 6.1			
VCO_2_, ml/kg/min					
Rest	2.7 ± 0.4	2.9 ± 0.6	0.515	<0.001	0.360
Stage 1	5.0 ± 0.5	4.8 ± 0.7			
Stage 2	8.5 ± 0.8	8.1 ± 0.7			
Stage 3	16.9 ± 3.2	16.8 ± 2.1			
RER					
Rest	0.88 ± 0.05	0.88 ± 0.06	0.945	<0.001	0.811
Stage 1	0.85 ± 0.05	0.85 ± 0.05			
Stage 2	0.92 ± 0.04	0.91 ± 0.04			
Stage 3	0.99 ± 0.07	1.00 ± 0.04			
HR, bpm					
Rest	60 ± 13	53 ± 10	0.056	<0.001	0.284
Stage 1	70 ± 16	70 ± 11			
Stage 2	81 ± 16	80 ± 10			
Stage 3	112 ± 16	107 ± 15			
RPE					
Rest	6 ± 1	7 ± 1	0.661	<0.001	0.150
Stage 1	8 ± 1	7 ± 1			
Stage 2	9 ± 2	9 ± 1			
Stage 3	12 ± 2	13 ± 1			
Body temp, °C					
Rest	35.3 ± 0.4	35.2 ± 0.5	0.795	0.033	0.352
Stage 1	35.4 ± 0.4	35.3 ± 0.5			
Stage 2	35.4 ± 0.3	35.4 ± 0.5			
Stage 3	35.4 ± 0.4	35.4 ± 0.5			

*Note*: Data are presented as mean ± standard deviation. *p* values are calculated by the repeated two‐way analysis of variance.

Abbreviations: ANOVA, analysis of variance; Body temp, body temperature; HR, heart rate; LC, land‐based cycling; RER, respiratory exchange ratio; RPE, Borg rating of perceived exertion; VCO_2_, carbon dioxide production; VO_2_, oxygen uptake; WC, water‐based cycling.

### 3.2 Breathing pattern

**FIGURE 2:**
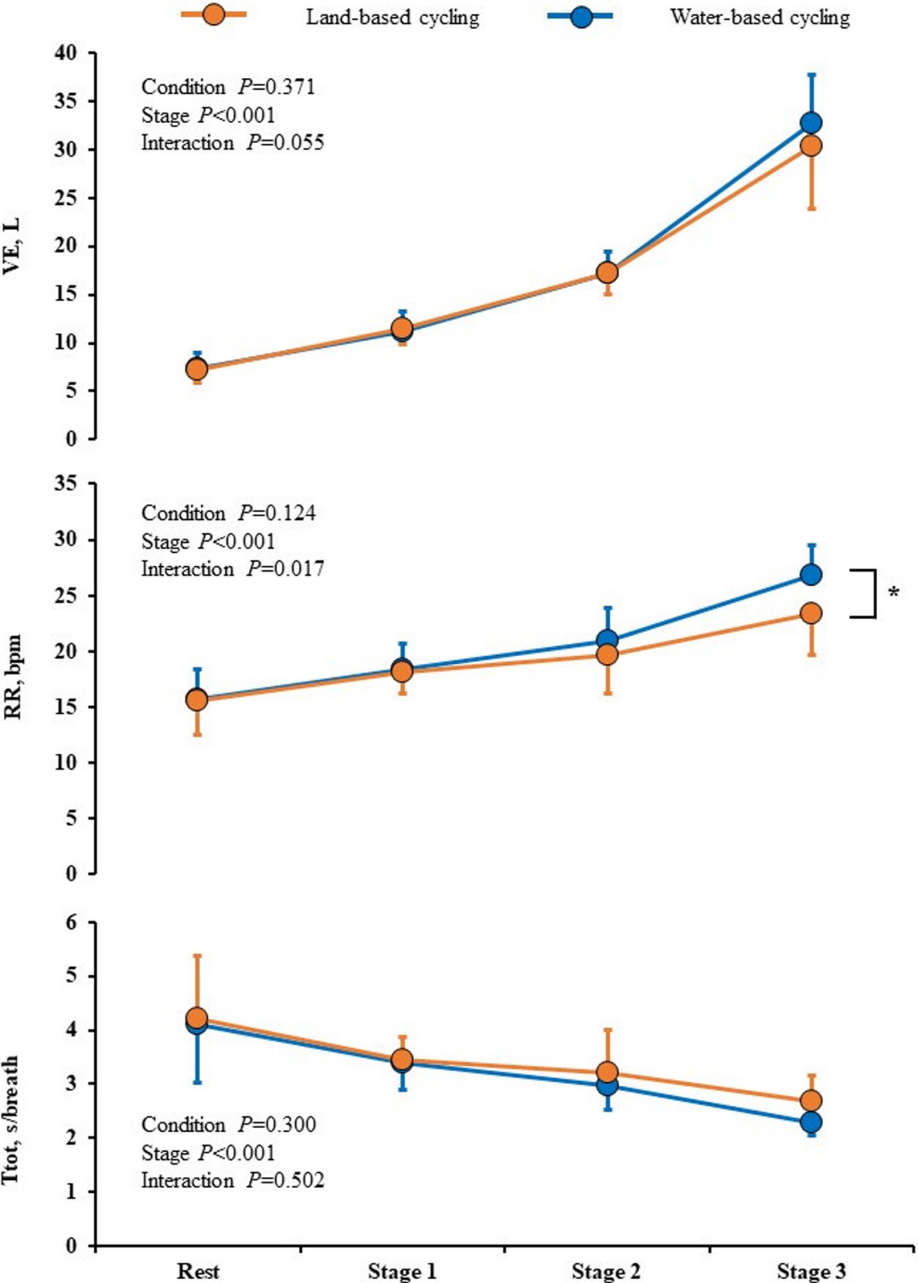
Ventilation (VE), respiratory rate (RR), and total breathing time (Ttot) during rest and cycling on land and in water. Orange and blue circles indicate the mean values measured in the land (LC)‐ and water (WC)‐based cycling conditions, respectively. Error bars indicate standard deviation (SD). *p* values are calculated by the repeated measures of two‐way analysis of variance. *p < 0.05, the difference between LC and WC.

**FIGURE 3:**
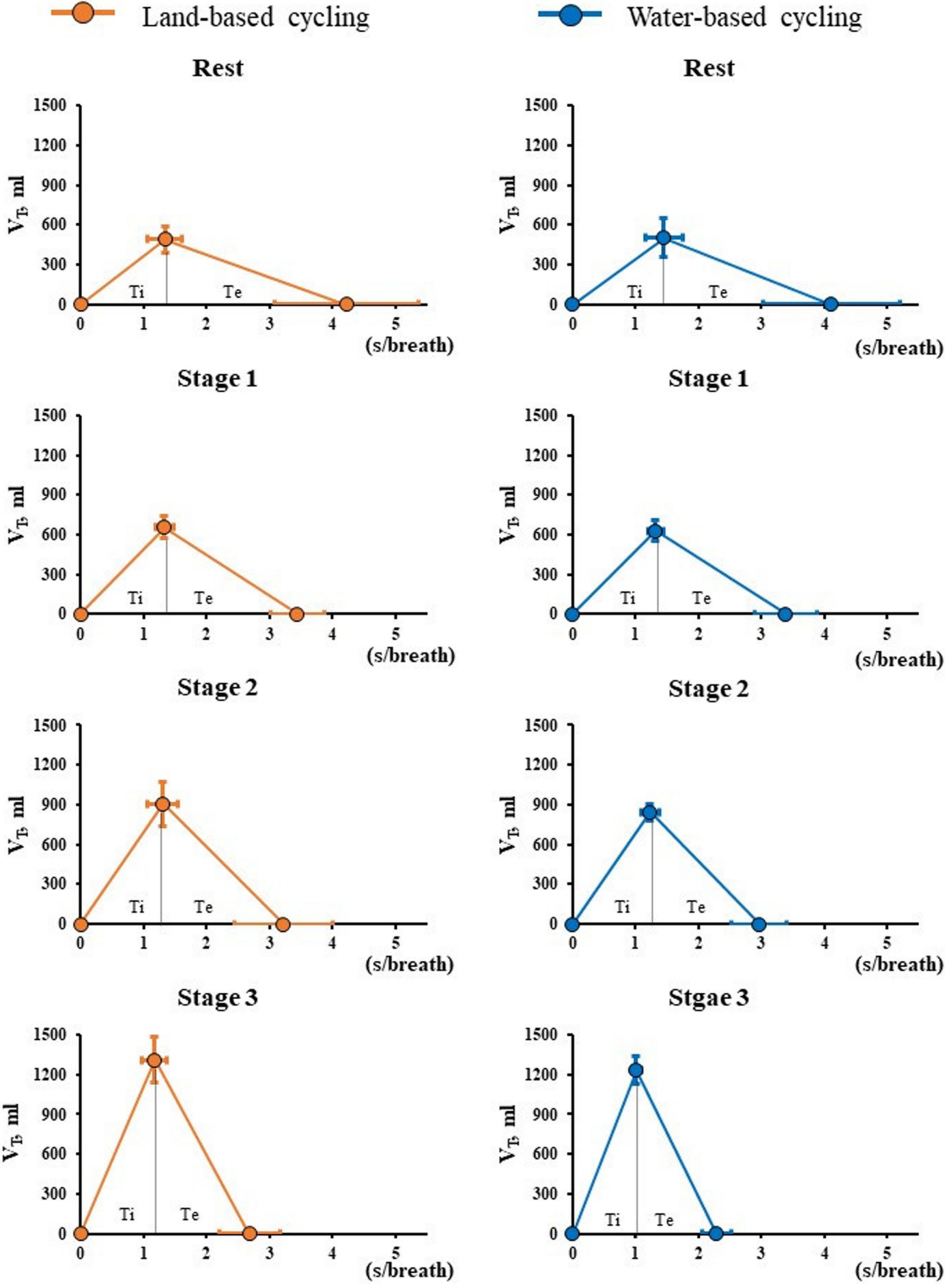
The relation between tidal volume and respiratory time per breath during rest and cycling on land and in water. Orange and blue circles indicate the mean values measured during land (LC)‐ and water‐(WC) based cycling, respectively. Error bars indicate standard deviation (SD). Statistical results and numeric data for this figure are presented in Table 2. V_T_, tidal volume which is the average of the inspiratory and expiratory phases; Ti, inspiratory time; Te, expiratory time.

**FIGURE 4:**
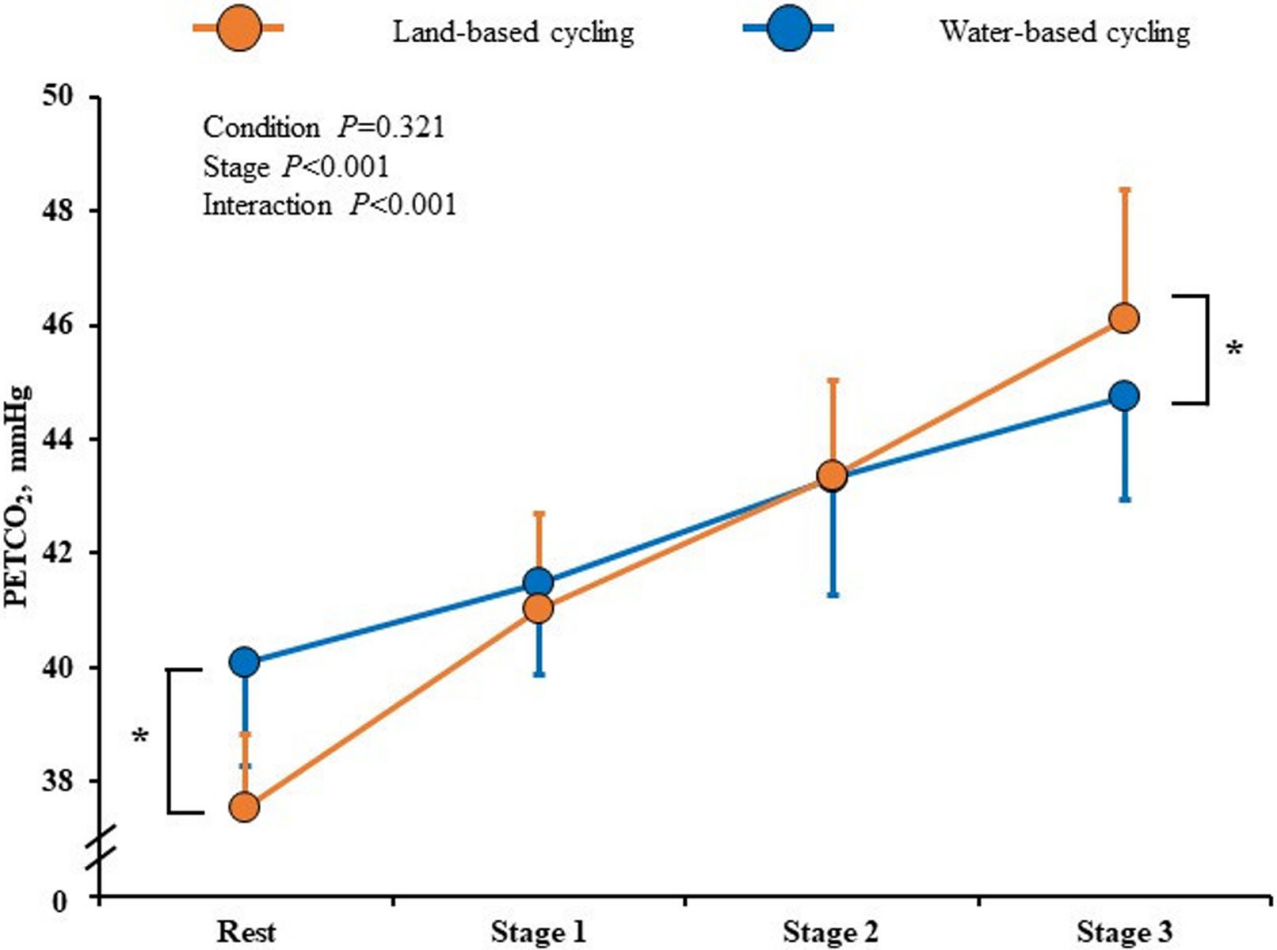
End‐tidal CO_2_ (PETCO_2_) during rest and cycling on land (LC) and in water (WC). Orange and blue circles indicate the mean in LC and in WC, respectively, and error bars indicate standard deviation (SD). *p* values are calculated by the repeated measures of two‐way analysis of variance. *<0.05, the difference between LC and WC.

In both conditions, all breathing variables changed significantly as VO_2_ increased. A similar magnitude of VE elevation and Ttot reduction was observed in WC and LC conditions. However, RR showed significant interaction. The *post hoc* test showed that RR was higher at stage 3 in WC than in LC (26.8 ± 2.7 vs. 23.3 ± 3.7 bpm, *p* = 0.012, Figure 2). Figure 3 and Table 2 show the relationship between tidal volume and respiratory time per breath, separately presented for the inspiratory and expiratory phases. In the expiratory phase, V_TE_ and expiratory flow showed similar increases, and Te showed similar decreases with increasing stages in both conditions. On the other hand, in the inspiratory phase, a significant interaction was observed in Ti and inspiratory flow, but not V_TI_. The result of the *post hoc* test showed that inspiratory flow was higher at stage 3 in WC than in LC, and Ti was lower in WC than in LC (Table 2). Moreover, PETCO_2_ showed significant interaction and was higher at rest (40.1 ± 1.8 vs. 37.6 ± 1.3 mmHg, *p* < 0.001) and lower at stage 3 (44.8 ± 1.8 vs. 46.1 ± 2.2 mmHg, *p* = 0.011) during WC than in LC (Figure 4).

**TABLE 2:** Tidal volume, breathing time, and respiratory flow divided into inspiratory and expiratory phase during rest and cycling on land and in water

Stage	LC	WC	*p* value (ANOVA)		
			Condition	Stage	Interaction
Inspiratory					
V_TI_, ml					
Rest	492 ± 94	509 ± 144	0.492	<0.001	0.303
Stage 1	649 ± 83	638 ± 85			
Stage 2	899 ± 161	855 ± 63			
Stage 3	1301 ± 164	1242 ± 107			
Ti, s/breath					
Rest	1.3 ± 0.3	1.5 ± 0.3	0.633	<0.001	0.007
Stage 1	1.3 ± 0.1	1.3 ± 0.1			
Stage 2	1.3 ± 0.2	1.2 ± 0.1			
Stage 3	1.2 ± 0.2	1.0 ± 0.1*			
Inspiratory flow, ml/s					
Rest	375 ± 79	350 ± 68	0.455	<0.001	0.006
Stage 1	491 ± 69	486 ± 74			
Stage 2	695 ± 71	700 ± 79			
Stage 3	1133 ± 200	1233 ± 173*			
Expiratory					
V_TE_, ml					
Rest	488 ± 97	500 ± 146	0.177	<0.001	0.124
Stage 1	658 ± 83	621 ± 76			
Stage 2	910 ± 170	833 ± 63			
Stage 3	1318 ± 184	1224 ± 105			
Te, s/breath					
Rest	2.9 ± 0.9	2.7 ± 0.8	0.212	<0.001	0.622
Stage 1	2.1 ± 0.3	2.1 ± 0.4			
Stage 2	1.9 ± 0.5	1.7 ± 0.3			
Stage 3	1.5 ± 0.3	1.3 ± 0.2			
Expiratory flow, ml/s					
Rest	179 ± 42	195 ± 48	0.309	<0.001	0.177
Stage 1	316 ± 41	309 ± 59			
Stage 2	491 ± 89	494 ± 83			
Stage 3	907 ± 228	979 ± 168			

*Note*: Data are presented as mean ± standard deviation. *p* values are calculated by the repeated two‐way analysis of variance. *vs. land in the same stage.

Abbreviations: ANOVA, analysis of variance; LC, land‐based cycling; Te, expiratory time; Ti, inspiratory time; V_TE_, tidal volume during expiratory; V_TI_, tidal volume during inspiratory; WC, water‐based cycling.

### 3.3 Respiratory function and maximal respiratory muscle pressure

Table 3 depicts the respiratory function and maximal respiratory muscle pressure before and after cycling exercise. The FVC and FEV_1_ showed significant interactions (*p* = 0.041 and 0.042, respectively). The post hoc test revealed that FVC was significantly decreased after WC (*p* = 0.010). On the other hand, no significant differences were observed for the FEV_1_, PEF, and FEV_1_/FVC. Moreover, the PImax and PEmax did not show a significant difference.

**TABLE 3:** Spirometric data before and after water‐based and land‐based cycling exercise

	Time	LC	WC	*p* value (ANOVA)		
				Condition	Time	Interaction
FVC, L	Pre	4.71 ± 0.42	4.80 ± 0.38	0.611	0.021	0.041
	Post	4.70 ± 0.39	4.67 ± 0.41*			
	Delta	−0.01 ± 0.10	−0.13 ± 0.14			
FEV_1_, L	Pre	4.07 ± 0.39	4.14 ± 0.35	0.496	0.955	0.042
	Post	4.12 ± 0.38	4.10 ± 0.39			
	Delta	0.05 ± 0.10	−0.05 ± 0.14			
FEV_1_/FVC, %	Pre	87.3 ± 5.8	87.1 ± 5.5	0.417	0.003	0.107
	Post	88.4 ± 6.1	89.3 ± 6.5			
	Delta	1.01 ± 1.66	2.18 ± 2.02			
PEF, L/s	Pre	9.80 ± 1.78	9.88 ± 1.43	0.747	0.445	0.182
	Post	9.80 ± 2.09	9.59 ± 1.78			
	Delta	−0.01 ± 0.61	−0.30 ± 0.86			
PImax, cmH_2_O	Pre	47.9 ± 15.7	47.4 ± 12.6	0.686	0.238	0.433
	Post	44.5 ± 13.4	46.8 ± 15.8			
	Delta	−3.4 ± 8.4	−0.6 ± 7.9			
PEmax, cmH_2_O	Pre	112.6 ± 18.4	110.0 ± 20.1	0.244	0.425	0.639
	Post	116.6 ± 26.0	111.5 ± 20.7			
	Delta	4.0 ± 14.1	1.5 ± 15.1			

*Note*: Data are presented as mean ± standard deviation. *p* values are calculated by the repeated two‐way analysis of variance. *vs. pre in the same condition.

Abbreviations: ANOVA, analysis of variance; FEV_1_, forced expired volume in one second; FVC, forced vital capacity; LC, land‐based cycling; PEF, peak expiratory flow; PEmax, maximal expiratory muscle pressure; PImax, maximal inspiratory muscle pressure; WC, water‐based cycling.

## 4 DISCUSSION

Using semi‐recumbent stationary bicycles, we were able to match VO_2_ during WC and LC and compare their respiratory and breathing responses. Our main findings from this study are as follows. First, ventilation and tidal volume were similar across all stages in both conditions. However, at moderate intensity, faster and shallower breathing was observed during WC than LC. Second, the decrease in vital capacity after WC was greater than LC. These findings suggest that underwater exercise performed at moderate intensity may place a greater load on the respiratory system than dryland exercise and may have the potential for respiratory muscle training and rehabilitation when compared with land‐based exercise. Below we discuss the novel aspects, potential mechanisms, and clinical implications of our findings.

### 4.1 Breathing pattern comparisons

Only a few studies have compared the breathing patterns during WC and LC. Sheldahl et al. performed incremental cycling exercise on land and in water at the intensities of 40%, 60%, and 80% VO_2_max and reported that respiratory rate was higher in water condition than on land condition at the 40% and 80% VO_2_max (Sheldahl et al., 1987). Consistently, our results showed that the respiratory rate was higher during WC than LC at moderate intensity. In addition, we found that the increase in respiratory rate was due to the reduction in inspiratory time rather than expiratory time. During water immersion, elevated hydrostatic pressure compresses the thoracic and abdominal cavities, causes the vertical displacement of the diaphragm, and restricts the expansion of the thorax (Agostoni et al., 1966). Moreover, increased hydrostatic pressure promotes venous return, which shifts the blood from the lower limbs to the chest and decreases lung compliance (Bréchat et al., 2013; Buono, 1983; Pendergast & Lundgren, 1985). A previous study conducted by our laboratory using the same experimental protocol found higher stroke volume during WC than LC at rest and during cycling (Fukuie et al., 2021). This suggests that our experimental setup was effective for increasing hydrostatic pressure which augments venous return due to the cephalad blood redistribution. These physiological changes caused by water immersion have been shown to increase the work of breathing (Pendergast et al., 2015) and decrease vital capacity (de Andrade et al., 2014). Therefore, water immersion may alter lung expansion (i.e., inspiration) which may be related to the reduction of inspiratory time.

Although we hypothesized that inspiratory time and volume would decrease during WC compared with LC, the results of this study did not show a decrease in inspiratory volume. In a previous study, the reduction of tidal volume occurred with higher intensity exercise (approximately 80% VO_2peak_) (Sheldahl et al., 1987). In addition, a previous study reported that water immersion decreased vital capacity by about 100–200 ml (de Andrade et al., 2014; Hoshi et al., 2021). However, tidal volume during moderate WC in this study was about 1250 ml, and hydrostatic pressure may not have much effect on this volume. Thus, a decrease in tidal volume may not have been observed at low‐to‐moderate‐intensity underwater exercise in the present study.

PETCO_2_ was higher in WC than LC at rest. In previous studies, PETCO_2_ at rest has also been shown to increase during water immersion (Sackett et al., 2017; Sackett et al., 2018). Water immersion increases transthoracic pressure, which increases the work of breathing (Pendergast & Lundgren, 1985). Moreover, elevated central blood volume increases dead space in the lungs (Bondi et al., 1976). These phenomena can cause hypoventilation, contributing to CO_2_ retention and higher PETCO_2_ (Cherry et al., 1985; Pendergast et al., 2015). On the other hand, there was no significant difference between low‐intensity LC and WC (14%–23% VO_2peak_), and the WC showed lower PETCO_2_ at stage 3 (approximately 43% VO_2peak_) than LC. At stage 3, PETCO_2_ may have decreased in WC due to increased respiratory rate during immersion in water.

In previous studies, hyperthermia and cold water immersion have been reported to increase ventilation (Fujimoto et al., 2016; Tsuji et al., 2016). Although core temperature such as esophageal temperature was not assessed in this study, the water temperature was maintained thermoneutral (31–32°C), which probably had a small impact on the metabolic system (Craig Jr. & Dvorak, 1966), and the tympanic temperature was not significantly different in both conditions. Thus, our results suggest that the increased respiratory rate in this study was not due to increased body temperature.

### 4.2 Respiratory function and respiratory muscle pressure

Yamashina et al. performed 30 minutes of water‐based and land‐based exercise at 60% VO_2peak_ and measured the respiratory function and muscle strength before and after exercise. Significant decreases in FEV_1_ and inspiratory and expiratory muscle pressures were observed after exercise in water conditions compared with land conditions (Yamashina et al., 2016). In this study, FVC showed significant decreases after WC. On the other hand, respiratory muscle pressure was not significantly different. Elevated hydrostatic pressure places an extra load on the respiratory system and increases the work of breathing. This consequently may cause respiratory muscle fatigue (Bréchat et al., 2013; Hoshi et al., 2021; Pendergast et al., 2015). However, the decreased spirometry values observed in our study may not only reflect respiratory muscle fatigue. Previous research observed an increase in unstable thoracic fluid balance similar to a preliminary stage of interstitial pulmonary edema during aquatic exercise (Bréchat et al., 2013). In fact, acute pulmonary edema has been reported during aquatic sports even in shallow water depth such as swimming and jogging (Koehle et al., 2005; Wenger & Russi, 2007). During water immersion, the pressure gradient between the pulmonary interstitial pressure and the intravascular pressure increases because of the increased blood volume associated with hydrostatic pressure (Gabrielsen et al., 1993; Younes et al., 1987). The repeated inspiratory load during exercise may facilitate the movement of plasma into the interstitium. However, all participants in the current study were healthy adults and did not complain of dyspnea after underwater exercise.

### 4.3 Clinical implications

Our results suggest possible clinical applicability of water‐based exercise for respiratory rehabilitation. The higher inspiratory flow showed at moderate‐intensity exercise during WC than LC, as accompanied by similar tidal volume during WC and LC and a shorter inspiratory time during WC than LC. In terms of respiratory muscle training, respiratory rate, tidal volume, and inspiratory flow would reflect the number of contractions, the contractility, and the contraction speed of inspiratory muscles, respectively. Therefore, our findings suggest that underwater exercise at moderate intensity may increase the number and speed of respiratory muscle contractions compared with land‐based exercise. Moreover, Yamashina et al. conducted an acute intervention experiment with 40 minutes of aquatic walking (60% VO_2_peak) and reported a significantly greater decrease in the maximal inspiratory muscle pressure at post‐exercise when compared with the land‐based walking condition (Yamashina et al., 2016). It has also been demonstrated that an underwater breathing exercise program improves respiratory muscle and spirometry values in older adults and patients with chronic obstructive pulmonary disease (Felcar et al., 2018; Ide et al., 2005). In the current study, changes in ventilatory response were only seen in stage 3 (43% VO_2_peak) during WC. Therefore, a higher tidal volume against increased hydrostatic pressure during underwater exercise may produce greater training effects than land‐based exercise performed at a similar intensity.

There are several limitations to this study. First, this study did not measure external power output during WC. Also, a pedaling cadence was different between the LC and WC conditions except for stage 3. Because the ventilatory function is influenced by various sensory inputs including afferent signals from locomotor muscles (Shevtsova et al., 2019), the differences in external power output may have altered RR independently from exercise environments (land vs. water). Previous studies have reported that the ventilatory response to locomotion appears at the onset of exercise and gradually adapts to the metabolic rate (Duffin, 2014; Koehle & Duffin, 1996). Because we collected ventilatory data during the last 30 s of the 5‐min stages, the confounding effects of external power output may be small, but it was impossible to determine the relative contributions in the current study. Thus, future studies comparing ventilatory responses during the WC and LC conditions need to consider matching external power output in addition to metabolic rate. Second, this study was conducted on healthy male subjects; therefore, whether the results of this study apply to women or different age groups is unclear. Older adults have decreased lung compliance associated with aging (Sharma & Goodwin, 2006), and thus an additional decrease in lung compliance associated with water immersion may place a greater burden and/or elicit adaptation in their respiratory system than younger adults. On the other hand, ventilation parameters and spirometry values vary widely depending on age and sex. This physiological variability could have masked the cardiopulmonary responses to exercise. Third, the order of WC and LC conditions was not randomized in order to match the metabolic rate of WC to LC. In this study, it was necessary to quantify the metabolic rate of each stage during WC and then adjust the VO_2_ during LC.

## 5 CONCLUSIONS

The breathing patterns during low‐intensity exercise did not differ between the underwater and dryland conditions. However, at a moderate intensity, respiratory rate and inspiratory flow increased during water‐based exercise due to shorter inspiratory time than those during land‐based exercise. These findings suggest that water‐based exercise at the same metabolic rate may have a greater impact on the respiratory system than land‐based exercise.

## AUTHOR CONTRIBUTIONS

All authors were responsible for the conception and design of this work. Daisuke Hoshi, Marina Fukuie, and Tatsuya Hashitomi performed experiments. Daisuke Hoshi analyzed data, prepared figures and tables, and drafted the manuscript. All authors edited and revised the manuscript and approved the final version of the manuscript and agree to be accountable for all aspects of the work in ensuring that questions related to the accuracy or integrity of any part of the work are appropriately investigated and resolved.

## FUNDING INFORMATION

This work was supported by the Japanese Society for the Promotion of Science (20K11303, KW).

## CONFLICT OF INTEREST

The authors declare that they have no potential conflict of interest.
